# Acute Sleep Deprivation and the Autoimmune TLR-BANK1 Pathway: Interplay with Gender and Emotional State

**DOI:** 10.3390/ijms27010375

**Published:** 2025-12-29

**Authors:** Marta Ditmer, Agata Gabryelska, Aleksandra Tarasiuk-Zawadzka, Agata Binienda, Szymon Turkiewicz, Filip Franciszek Karuga, Aleksandra Wojtera, Piotr Białasiewicz, Jakub Fichna, Dominik Strzelecki, Marcin Sochal

**Affiliations:** 1 Department of Sleep Medicine and Metabolic Disorders, Medical University of Lodz, 92-215 Lodz, Poland; 2 Department of Biochemistry, Medical University of Lodz, 92-215 Lodz, Poland; 3 Department of Affective and Psychotic Disorders, Medical University of Lodz, 92-216 Lodz, Poland

**Keywords:** BANK1, sleep, sleep deprivation, TLR7, TLR9

## Abstract

Deprivation of sleep (DS) is linked to increased risk of immune-mediated diseases. Toll-like receptors (TLR7, TLR9) and BANK1 are key B-cell signaling components that may contribute to their pathogenesis. Seventy-six adults underwent polysomnography (PSG) followed by DS. Venous blood was collected after PSG and DS. Mood was evaluated before and after each stage using Montgomery–Åsberg Depression Rating Scale. Participants were classified as Responders (REs) or Non-Responders (NRs) based on mood changes post-DS. Gene mRNA expression of TLR7, TLR9, and BANK1 in peripheral blood mononuclear cells was analyzed by qRT-PCR. DS reduced TLR7 expression in the entire study group and within NRs, REs, and male and female subgroups (all *p* < 0.001). During analysis of covariance, women exhibited higher TLR7 expression than men post-DS (*p* = 0.022), independent of age and body mass index (BMI). At baseline, women exhibited lower expression of TLR9 (*p* = 0.009, independent of age and BMI), which was abolished after DS (*p* = 0.570). BANK1 expression increased post-DS in the entire study group and in NRs (*p* = 0.021), but not REs (*p* = 0.329). DS modulates B-cell-related immune signaling, with reduced TLR7 and increased BANK1 expression in a sex- and mood-dependent manner.

## 1. Introduction

Insufficient sleep has become a pervasive issue in modern industrialized societies [1]. Shift work, one of the main causes of induced sleep disorders, is associated with several immunological diseases, including inflammatory bowel disease or systemic lupus erythematosus (SLE). It has also been connected to poor mental health [2]. Despite growing awareness of these consequences, the biological mechanisms linking altered circadian patterns to immune dysregulation remain incompletely understood. A key factor under investigation is deprivation of sleep (DS), which refers to the externally (e.g., work schedules, environmental noise) or internally (e.g., chronic pain) imposed restriction or elimination of sleep.

The consequences of DS are wide-ranging including, among others, elevated oxidative stress and disturbances in the expression of genes that regulate circadian rhythms [3,4]. Another proposed pathway involves the circadian regulation of Toll-like receptors (TLRs)—innate sensors implicated in immune responses. DS-induced disruptions in clock gene expression may alter TLR expression, potentially leading to inappropriate immune activation. TLR7 and TLR9 are of particular interest because of their ability to recognize endogenous nucleic acids: TLR7 detects single-stranded RNA, whereas TLR9 recognizes unmethylated CpG DNA, thereby promoting B-cell activation and subsequent autoantibody production [5,6].

TLR9 is localized in endosomes of macrophages, dendritic cells, natural killer cells, and B-cells, where its activation triggers the production of proinflammatory cytokines and interferon-γ [7]. In murine models, TLR9 has been shown to promote the generation of anti-DNA plasmablasts, and mice lacking TLR9 exhibit impaired anti-double stranded DNA (dsDNA) antibody production [8,9]. In human studies, TLR9 is upregulated in patients with SLE and active ulcerative colitis [10,11]. Interestingly, although clinical data often suggest a pathogenic role, animal models indicate that TLR9 may exert a protective effect, especially in terms of tissue repair and modulation of inflammation [11]. This paradox suggests that a balanced expression of TLR9 may be necessary to maintain immune homeostasis.

In contrast, TLR7, which shares structural and functional similarities with TLR9, is suggested to have a more clearly pathogenic role in immune-mediated disorders. It stimulates the secretion of proinflammatory cytokines (e.g., IL-6, TNF (tumor necrosis factor)), type I interferons, and immunoglobulins, particularly IgG and IgM [12,13]. While not required for anti-dsDNA antibody production, TLR7 is essential for antibody responses against RNA-associated antigens, and it has been implicated in models of experimental autoimmune encephalomyelitis and severe SLE [10,14,15]. However, its role in other such conditions remains ambiguous. Notably, some evidence suggests that TLR7 deficiency increases susceptibility to colitis, highlighting the complexity of its immune functions [16].

An additional layer of regulation involves BANK1 (B-cell scaffold protein with ankyrin repeats 1)—a scaffold protein that integrates signals from B-cell receptors (BCR), CD40, and TLR pathways [17,18]. BANK1 contains a Toll/IL-1 receptor (TIR) domain that interacts with the adaptor protein myeloid differentiation primary response 88 (MYD88), initiating downstream signaling cascades involving interleukin-1 receptor-associated kinase 1 (IRAK-1), tumor necrosis factor receptor-associated factor 6 (TRAF6), nuclear factor kappa-light-chain-enhancer of activated B-cells (NF-κB), and interferon regulatory factor 7 (IRF7) [18]. These pathways culminate in the production of type I interferons and proinflammatory cytokines, including IL-6, which supports B-cell differentiation and immunoglobulin production [17,19]. BANK1 has recently gained attention for its role in autoimmunity: BANK1-deficient mice show reduced levels of anti-dsDNA autoantibodies and decreased IL-6 production [20,21]. Furthermore, in collagen-induced arthritis models, lower BANK1 expression was associated with milder disease and fewer autoantibodies [22].

Notably, women are more frequently affected by both sleep disorders, such as insomnia, and autoimmune conditions, highlighting the potential influence of sex in this context [23,24].

Sex-based differences in immune responses might contribute to the higher prevalence of immune-mediated disorders in women [24]. TLR7, located on the X chromosome and capable of escaping X inactivation, is expressed at higher levels in women, enhancing immune response and reducing susceptibility to infections compared to men [24]. TLR9 activity may be modulated by estrogens [25]. While data on sex differences in BANK1 expression are limited, its regulation by upstream mediators supports the plausibility of such distinctions.

The aim of the present study was to investigate the effects of DS on the changes in mRNA expression levels of TLR7, TLR9, and BANK1, accounting for differences in the expression of these genes between men and women as well as different emotional responses to sleep loss.

We hypothesize that DS increases the expression of TLR7, TLR9, and BANK1, with these effects being more pronounced in women compared to men as well as in individuals whose depressive symptoms worsened after a sleepless night.

## 2. Results

### 2.1. Baseline Data Regarding the Study Group and Subgroups

Of 113 recruits, 37 were excluded: 31 for incomplete protocol, 5 for total sleep time < 5 h, and 1 for missing data regarding the expression of the studied genes, leaving 76 participants for analysis. Baseline demographic characteristics are presented in Table 1.

In the entire study group, the median age was 24 years (interquartile range (IQR): 22–26 years, Table 1) and the median BMI was 22.7 (IQR: 21.1–24.8, Table 1). There were no significant differences between REs and NRs regarding the collected demographic data (all *p* > 0.05, Table 1). Female participants were significantly younger and exhibited lower BMI compared to males (*p* = 0.017 and *p* = 0.006 for age and BMI, respectively; Table 1). This article is a part of a larger scientific project; details regarding baseline PSG parameters and actigraphy are available in the previous report [26].

### 2.2. DS-Induced Changes in the Context of Sex

Comparison between the morning after PSG and after DS within the entire study group revealed that DS significantly decreased the expression levels of TLR7 (*p* < 0.001; Table 2).

Downregulation of TLR7 expression was observed in both male and female subgroups (all *p* < 0.001; Table 2). ANCOVA indicated that women and men had comparable baseline TLR7 expression; however, following DS, women displayed significantly higher TLR7 expression than men (*p* > 0.05 after PSG, *p* = 0.022 after DS independently of age and BMI; Table 2).

DS did not affect the expression of TLR9 in the entire study group or within the male and female subgroups (all *p* > 0.05; Table 2). In an ANCOVA, women demonstrated lower expression of TLR9 than men at baseline assessment, but not after DS (*p* = 0.009 after PSG, independent of age and BMI, *p* > 0.05 after DS; Table 2).

In the entire study group, BANK1 expression was significantly higher after DS compared with the morning after PSG (*p* = 0.031; Table 2). No sex differences in BANK1 expression were observed at any time point (*p* > 0.05; Table 2).

### 2.3. DS-Induced Changes in the Context of Mood Regulation

Both NR and RE groups showed decreased TLR7 expression after DS (*p* < 0.001 for NR and RE; Table 3), while TLR9 expression remained unchanged (*p* > 0.05; Table 3) compared to the baseline values.

In the NR group, BANK1 expression increased significantly after DS relative to baseline, whereas no change was observed in the RE group (*p* = 0.021 and *p* > 0.05, respectively; Table 3). No significant differences in the expression of any studied genes were detected between REs and NRs either at baseline or post-DS (*p* > 0.05; Table 3).

## 3. Discussion

The current literature offers limited insight into the impact of DS on immunity; even less is known about its interactions with mood. The demanding nature of experimental protocols often poses challenges for participant recruitment, making it difficult to obtain sufficient groups for comparisons between their subsets created based on sex, mood response to DS, etc. To the best of our knowledge, this is the first study to investigate the effects of DS on the mRNA expression of selected genes associated with B-cell signaling, while also exploring their relationship with mood changes and sex-related differences.

One of the main findings of this study is that DS downregulated TLR7 expression in the entire study group. The only other available study on the influence of sleep deficiency on TLR7 was conducted using a model of prolonged sleep restriction—4 h of sleep per night for 5 days—in a small group of men (*n* = 9) [27]. Authors observed the opposite results compared to the present study, namely, an approximately 1.5-fold increase in TLR7 expression [27]. However, using a model of prolonged sleep restriction instead of acute DS may lead to different alterations in the expression of circadian clock genes, as demonstrated for Period 1 (PER1). While insomnia has been associated with decreased expression of PER1, acute DS may increase its expression [4,28]. Other circadian rhythm-regulating genes could show similar divergent patterns depending on the type of sleep insufficiency, which in turn may influence TLR7 expression [4,28]. Nonetheless, the relationship between this receptor and the circadian rhythm remains unclear. Greenberg et al. showed that another clock gene, Brain and Muscle ARNTL-Like 1 protein (BMAL1), is able to regulate TLR7-mediated immune response in the skin, thus implying the existence of interactions between the two [29].

It appears that TLR7 expression is related to both age and BMI. In a study by Kanaan et al., TLR7 gene copy number increased with age in PBMCS, while Sindhu et al. demonstrated that TLR7 expression in adipocytes is positively correlated with BMI [30,31]. Thus, the present study revealed that although baseline TLR7 expression did not differ between women and men, DS led to higher TLR7 expression in women compared with their male counterparts, independent of age and BMI. No studies to date have analyzed sex-specific differences in TLR7 expression in PBMCs. However, in the light of the current knowledge, those findings appear counterintuitive. It would be expected that women would consistently exhibit higher expression of TLR7, since the location of this gene on the X chromosome is capable of escaping X-inactivation, thereby resulting in elevated expression in female cells [24]. Nonetheless, TLR7 expression is known to vary among immune-cell populations, with particularly prominent expression in B-cells [32]. Therefore, bulk mRNA analysis in PBMCs may not have been sensitive enough to detect cell-type-specific sex differences in TLR7 expression at baseline assessment.

The observed sex difference in TLR7 expression, together with the overall reduction in TLR7 levels after DS, suggests sex-specific immunomodulatory effects of DS. Elevated TLR7 expression in women has long been proposed to contribute both to their enhanced responses to infection and their increased susceptibility to autoimmune diseases [24,33]. Supporting this notion, Miles et al. demonstrated that female lung cells exhibit higher TLR7 expression than male cells, accompanied by a more robust immune response, including increased antibody production and elevated expression of proinflammatory cytokines and interferons [33]. These findings raise the possibility that women, already at higher baseline risk for autoimmune disorders, may be particularly vulnerable under conditions that impose DS, such as shift work.

As for TLR9, it was observed that the expression of this receptor was not affected by DS in the entire study group. There are no available studies analyzing the impact of DS on TLR9; however, considering its well-established relationship with circadian rhythm, such findings are counterintuitive. Silver et al. demonstrated that BMAL1: Circadian Locomotor Output Cycles Kaput (CLOCK) heterodimer is able to control the expression of TLR9 through binding to the E-boxes located near the transcription initiation site [7]. Taira et al. further confirmed those findings using an animal model [34]. It is worth noting that both of the aforementioned studies were conducted in animals; the circadian regulation of TLR9 expression may differ in humans. Re-examining the interactions between TLR9 and clock genes could yield new insights and expand the understanding of the immune system.

Similarly to TLR7, TLR9 expression also appears to be related to body weight and age. In a study by Wang et al., mice deficient in TLR9 in response to a high-fat diet exhibited greater adipose tissue inflammation, increased body weight, and reduced glucose and insulin tolerance compared to wild-type counterparts [35]. Letiembre et al. revealed that in the mouse brain, TLR9 expression decreased with age [36]. Thus, after adjusting for age and BMI as covariates, it was revealed that women exhibited higher baseline TLR9 expression than men, independent of the abovementioned factors; this difference was eliminated after DS. There were no differences in TLR9 expression between baseline and post-DS in either subgroup. Although no studies directly confirm lower peripheral TLR9 expression in women compared to men observed here after PSG, available evidence suggests that such difference is plausible. In a study on mice, both male and female TLR9 knock-out animals showed similar susceptibility to infection with mouse cytomegalovirus (MCMV) [37]. However, in wild-type mice, males exhibited higher expression of TLR9 in spleen compared with female counterparts [37]. Those changes appeared only after infection, spleens from uninfected mice exhibited uniformly low TLR7 and TLR9 mRNA expression, with no significant sex-related differences [37]. In a study by Koupenova et al., among subjects with normal BMI, platelet expression of TLR9 was similar between men and women, whereas TLR7 expression was higher in women, as expected based on the findings discussed above [38]. Estrogen levels also do not appear to explain such divergent results. Dennison et al. found that the responsiveness of TLR9 does not fluctuate across the menstrual cycle [39]. Cummingham et al. showed that activation of TLR9 in the spleen and dendritic cells relies on the estrogen receptor alpha (Erα) binding to the estrogen-responsive element; however, this process takes place independently of the presence of estrogen [40].

Following DS, TLR9 expression did not differ between sexes, suggesting a potential modification of mechanisms regulating its expression. Further research is warranted to identify upstream factors in TLR signaling that modulate ERα binding to estrogen response elements, particularly those responsive to DS [40].

TLR7 and TLR9 may fulfill distinct roles in immunity, potentially balancing each other’s effects. This interplay has important clinical implications, as it has been hypothesized that TLR9 exerts a protective function, whereas TLR7 plays a pathogenic role in the development of SLE [41]. This may be partially attributed to the apparent dual role of TLR9 in both immune tolerance and autoimmunity; while TLR9 facilitates the clearance of autoreactive cells in the periphery, it is also required for their activation [42]. In the present study, the observed reduction in TLR7 expression, together with relatively higher post-DS TLR7 expression in women and the absence of baseline sex differences in TLR9 expression following DS, may reflect sex-related differences in immune-related gene expression associated with sleep loss. Although the functional implications of these findings remain unclear, they may point to differential engagement of immune signaling pathways in men and women. Further studies are required to determine the biological and clinical relevance of these observations and to clarify whether such changes have implications for immune function or disease susceptibility.

In the present study, within the entire study group, DS increased the expression of BANK1; such effect was not present in the male or female group, likely due to small sample sizes. There were also no significant differences in BANK1 expression between men and women at any time point. No studies to date have examined BANK1 expression in PBMCs of either humans or animal models under DS conditions; however, sleep loss-induced increase in the expression of BANK1, alongside a decrease in TLR7, suggests the involvement of additional mediators that may promote this pathway. The absence of baseline differences between sexes may indicate that this signaling pathway exhibits comparable activity in men and women.

Depression and its pharmacological treatment have been shown to affect immunity, including TLR-related pathways [43]. Given that DS also disrupts immune function, these processes may plausibly modulate mood responses to acute lack of sleep. Acute lack of sleep is known to shift the cytokine profile toward a more proinflammatory state, partly through the upregulation of tumor necrosis factor (TNF) levels [44]. Notably, a study by Foo et al. demonstrated that depressed patients, particularly those who experienced symptomatic improvement following DS, exhibited lower TNF expression after DS compared with baseline values [45]. Since TLR7 was demonstrated to promote TNF production, these findings suggest a potential interplay between mood regulation and the mentioned elements of immune response under conditions of DS, warranting further investigation [46,47]. TLR7- and TLR9-induced proinflammatory cytokines are known to activate the kynurenine pathway, resulting in impaired tryptophan metabolism, decreased serotonin levels, and increased neurotoxic metabolites such as quinolinic acid, which is also induced by DS [48]. These mechanisms may play a key role in the development of depressive symptoms and the worsening of sleep disturbances [49].

In the present study, TLR7 expression decreased in both subgroups defined by the severity of depressive symptoms following acute DS, whereas TLR9 expression remained unchanged in these groups. These results suggest that TLR7 and TLR9 might not be key factors in the molecular mechanisms underpinning the interplay between DS, depressive symptoms, and immune function. Previous studies have indicated their involvement in mood regulation. In an animal model, imiquimod, a TLR7-specific ligand, promoted depressive behaviors in mice [50]. In another study, mice subjected to acute stress exhibited higher hippocampal expression of TLR7 and its downstream mediators, Myd88 and TRAF6; antidepressant treatment was able to reverse this effect [51]. Less is known about TLR9. However, in a study by Hung et al., patients with depression demonstrated higher expression of several TLRs, including TLR7 and TLR9 in PBMCs [52]. Antidepressant treatment subsequently decreased the expression of both discussed receptors [52]. Discrepancies in the results may stem from the inclusion of healthy participants in the present study, whereas other investigations involved individuals with diagnosed depression.

On the contrary, BANK1 expression increased in participants who experienced mood deterioration following DS, but not in those whose mood improved or remained stable. This suggests a potential role for BANK1 in linking DS to mood alterations. The literature on the relationship between BANK1 and depressive symptoms is limited. Current evidence suggests a potential association with brain volume and depression; however, these findings are relatively tenuous and have not been replicated in other studies [53,54]. Nevertheless, such observations indicate that subjects whose mood deteriorated after DS might exhibit a more robust activation of B-cell-related signaling pathways. This potentially could render the former particularly prone to the development of autoimmunity, providing another aspect to the discussion on the subject on interactions between immunity and mental health.

Future studies should focus on the role of BANK1 in mood and sleep disorders, aiming to identify other than TLR7 and TLR9 upstream regulators involved in this pathway. Investigating this relationship might elucidate the interplay between depressive symptoms, immunity, and inflammation, which would aid in identifying novel treatment targets for this disorder. Integrating molecular, behavioral, and clinical approaches will be essential to gain sufficient insight into the mechanisms underlying the impact of DS on the organism.

Several limitations of this study need to be acknowledged. Only gene expression at the mRNA level was assessed, without measuring the corresponding protein concentrations in serum. This restricts interpretation to the transcriptional phase of protein synthesis, especially limiting the insight into the biological availability and effects of BANK1. Consequently, the findings were not validated at the protein level, nor were downstream signaling pathways or functional outcomes examined. Future studies should address these aspects by incorporating protein-level validation, pathway analyses, and functional experiments to better elucidate the underlying biological mechanisms.

Nonetheless, this approach is well-suited for short-term DS protocols, where limited wake time may not allow for detection of post-transcriptional changes. Focusing on mRNA expression avoids confounding results with incomplete protein-level data. PSG was performed on a single occasion, introducing the potential influence of the “first night effect”. Additionally, the use of actigraphy may have failed to detect brief naps or misclassified periods of inactivity as sleep, particularly since DS occurred outside of a controlled sleep laboratory environment. While some DS studies adopt a constant routine protocol to minimize environmental influences, the current design allowed participants to maintain their activity during DS under normal conditions. Another important limitation is that individual participants were allowed to use medications which affect hormone levels (i.e., contraception, progesterone, spironolactone, levothyroxine). Female participants could also have experience menstrual cycle changes during the study, which could have influenced the results. Women also exhibited minor but significant differences regarding age and BMI compared to their male counterparts, which was accounted for by performing ANCOVA with the abovementioned parameters as covariates.

Findings described in the present study suggest a possible sex-specific association between DS and the expression of genes related to the TLR–BANK1 signaling pathway; however, the observed changes in women may reflect a greater engagement of pathways relevant to autoimmunity rather than a direct causal effect. Future studies should further clarify the mechanisms underlying DS-induced immune modulation. Such insights could inform strategies to mitigate the adverse effects of DS, particularly in high-risk groups such as shift workers.

## 4. Materials and Methods

### 4.1. Protocol

The study included individuals aged 18–35 years with a BMI of 20–30 kg/m^2^, who provided written informed consent. The following exclusion criteria were applied: pregnancy/lactation, chronic diseases (unstable endocrine/metabolic disorders, immune-mediated diseases, and renal, pulmonary, or cardiac insufficiency), radio/chemotherapy, malignant neoplasms with the exception of basal cell carcinoma, surgery within the last 6 months, substance abuse, diagnosis of sleep disorders within the last 2 years, infection, and intercontinental travel within two weeks prior to enrolment.

The study consisted of two phases: polysomnography (PSG) and DS. Participants were volunteers who provided written informed consent and had a physical examination prior to undergoing PSG. PSG was conducted in order to confirm normal sleep architecture. PSG recordings included electroencephalography (EEG) to monitor brain activity; electromyography (EMG) of the chin and anterior tibialis muscles to assess muscle tone; electrooculography (EOG) to track eye movements; a thermistor sensor to measure oronasal airflow; snoring detection; body position sensors to record sleep posture; piezoelectric belts to assess chest and abdominal respiratory effort; a unipolar electrocardiogram (ECG) to monitor cardiac activity; and pulse oximetry (SpO_2_) for oxygen saturation (Alice 6, Philips-Respironics, Murrysville, PA, USA). Sleep stages were scored in 30 s epochs according to American Academy of Sleep Medicine (AASM) standards.

A single night of DS was conducted under actigraphic monitoring (GENEActiv Original, ActivInsights Ltd., Cambs, UK) approximately 2–4 weeks after PSG. DS duration was set at ca. 24 h, from one morning to the next, with ca. 12 h of actigraphic monitoring. On the scheduled evening, participants were admitted to the Department of Sleep Medicine and Metabolic Disorders. Each participant was equipped with an actigraph and received detailed instructions regarding the DS protocol, including avoidance of naps and the use of psychoactive substances. Participants were allowed to spend the night in their place of residence. DS concluded the following morning at approximately 8:00 a.m.

Venous blood collection (9 mL) was conducted at two time points, in the morning after PSG or DS. Mood was assessed before and after each stage. Mood was assessed by a trained rater using the Montgomery–Åsberg Depression Rating Scale (MADRS), a 10-item instrument designed to evaluate depressive symptomatology. Total scores range from 0 to 60, with higher values indicating greater symptom severity, and encompass domains such as sadness, tension, and sleep disturbance. A MADRS score above 7 is considered clinically relevant and indicative of mild depression. Participants were categorized as Non-Responders (NRs), defined as those without overnight improvement in MADRS scores, or Responders (REs), defined as those who demonstrated an improvement in MADRS scores or maintained a stable score below 8 overnight.

Actigraphy was scored according to AASM guidelines [55]. Signal acquisition was standardized to 1 min epochs. Sedentary time was classified as periods with a gravity-subtracted vector-magnitude sum < 386 [56].

The study protocol was approved by the Bioethics Committee of the Medical University of Lodz (reference number: RNN/302/20/KE).

### 4.2. Molecular Analysis

Gene expression was analyzed at two time points (morning after PSG or DS). RNA was isolated via the Trizol method (Invitrogen, Waltham, MA, USA) and quantified with a spectrophotometer (Nanodrop Colibri, Titertek Berthold, Pforzheim, Germany). Then, cDNA was synthesized (SuperScript IV First-Strand Synthesis System, Thermo Fisher Scientific, Carlsbad, CA, USA). Quantitative reverse transcription polymerase chain reaction (qRT-PCR) was conducted using the Rotor-Gene™ 3000 thermal cycler (Corbett Research, Mortlake, NSW, Australia). The reaction mixture included BANK1, TLR7, and TLR9 probes (Taqman, Thermo Fisher Scientific, Carlsbad, CA, USA), the reference gene glyceraldehyde-3-phosphate dehydrogenase (GAPDH), nuclease-free water, a master mix reaction mixture, and cDNA. Each sample was tested in a triplicate, with the cycle threshold (CT) being calculated for each replicate. The results were presented as ΔCT and analyzed using the 2^−∆∆CT^ equation.

### 4.3. Statistical Analysis

Statistical analysis was performed using Statistica 13.1PL (StatSoft, Tulsa, OK, USA). A *p*-value < 0.05 was considered statistically significant. Gene expression data were log-transformed to correct for skewness. For each gene, expression indices were calculated as the ratio Δ = post-DS value/post-PSG value. The distribution of the variables was evaluated using the Shapiro–Wilk test. Data were presented as median with interquartile range or mean with standard deviation in case of non-normal and normal distribution, correspondingly. Parametric variables were compared using Student’s *t*-test, while non-parametric variables were analyzed with either the Wilcoxon signed-rank test or the Mann–Whitney U test, as appropriate. An analysis of covariance (ANCOVA) was performed with age and BMI as covariates in order to investigate the differences in gene expression between sexes. Effect size was estimated using partial eta-squared (η^2^) for ANCOVA, with values of 0.01, 0.06, and 0.14 indicating small, moderate, and large effects, respectively. For the Mann–Whitney U and Wilcoxon tests, rank-biserial correlation (*r_g_*) was applied, where values of 0.1, 0.3, and 0.5 represent small, medium, and large effects.

## Figures and Tables

**Table 1:** Baseline demographic characteristics.

	All	Women	Men	*p*-Value, Women vs. Men	NR	RE	*p*-Value, NR vs. RE
n, %	76	39, 51.3%	37, 48.7%	-	29, 38.2%	47, 61.8%	-
Women (n, %)	39, 51.3%	-	-	-	18, 62.1%	21, 44.7%	0.216
Men (n, %)	37, 48.7%	-	-	-	11, 37.9	26, 55.3%	
Age (years, median, IQR)	24 (22–26)	23 (22–26)	24 (23–26)	0.017	23 (22–25)	24 (23–26)	0.102
BMI (kg/m^2^, median, IQR)	22.7 (21.1–24.8)	22.15 (20.7–23.4)	24.30 (22.2–25.9)	0.006	22.60 (19.5–24.7)	22.88 (21.6–24.8)	0.215
Smoking (n, %)	9, 11.8%	4, 10.3%	5, 13.5%	0.933	4, 13.8%	5, 10.6%	0.962
Surgical operations (n, %)	29, 38.2%	13, 33.3%	16, 43.2%	0.514	9, 31.0%	20, 42.6%	0.447

Abbreviations: BMI—body mass index; IQR—interquartile range; n— number of participants, NR—Non-Responders; RE—Responders. Data are presented as n, median (interquartile range, (IQR)) due to non-normal distribution.

**Table 2:** Sex-dependent differences in expression of TLR7, TLR9, and BANK1 under the conditions of sleep deprivation.

		All Participants	*p*-Value All Participants, Post-PSG vs. All Participants Post-DS, Effect Size	Women	Men	*p*-Value Women, Post-PSG vs. Women, Post-DS, Effect Size	*p*-Value Men, Post-PSG vs. Men, Post-DS, Effect Size	*p*-Value Women, Post-PSG vs. Men, Post-PSG, Effect Size	*p*-Value Women, Post-DS, vs. Men, Post-DS, Effect Size
After PSG	BANK1	72, −3.5 ((−3.9)–(−2.7))	0.031 *r_g_* = 0.25 **	38, −3.2 ((−3.9)–(−2.6))	34, −3.7 ((−4.0)–(−3.2))	0.157	0.135	0.609 *	0.237 *
After DS		76, −3.1 ((−3.5)–(−2.8))		39, −3.0 ((−3.4)–(−2.8))	37, −3.1 ((−3.5)–(−2.8))				
After PSG	TLR7	75, −0.7 ((−1.0)–(−0.5))	<0.001 *r_g_* = 0.67 **	39, −0.6 ((−1.1)–(−0.3))	36, −0.8 ((−0.9)–(−0.6))	<0.001 *r_g_* = 0.63 **	<0.001 *r_g_* = 0.72 **	0.281 *	0.022 * η_p_^2^ = 0.07 **
After DS		76, −1.4 ((−2.1)–(−0.9))		39, −1.2 ((−2.0)–(−0.7))	37, −1.6 ((−2.2)–(−1.1))				
After PSG	TLR9	68, 0.6 (0.1–1.0)	0.523	36, 0.4 ((−0.0)–0.8)	32, 0.7 (0.4–1.1)	0.106	0.460	0.009 * η_p_^2^ = 0.10 **	0.570 *
After DS		76, 0.7 (0.1–1.2)		39, 0.7 (0.1–1.2)	37, 0.9 (0.0–1.2)				
						*p*-value, women vs. men			
ΔBANK1		72, 1.1 (0.9–1.3)		38, 1.1 (0.9–1.2)	34, 1.1 (0.9–1.3)	0.526 0.805 *			
ΔTLR7		75, 0.5 (0.3–1.0)		39, 0.5 (0.2–1.0)	36, 0.5 (0.3–0.9)	0.732 0.718 *			
ΔTLR9		68, 0.6 ((−0.1)–1.3)		36, 0.7 ((−0.1)–1.1)	32, 0.6 ((−0.1)–1.8)	0.869 0.744 *			

* Analysis of covariance (ANCOVA). Abbreviations: BANK1—B-cell scaffold protein with ankyrin repeats 1; DS—deprivation of sleep; PSG—polysomnography; TLR7/TLR9—Toll-like receptor 7/9; Δ—post-DS value/post-PSG value. Data are presented as n, median (interquartile range, (IQR)) due to non-normal distribution. Data marked with an asterisk (*) indicate the results of an ANCOVA, which was performed in order to account for baseline differences in age and BMI between male and female participants, with abovementioned variables as covariates. Double asterisks (**) denote effect sizes. Effect sizes were reported for statistically significant results as partial eta-squared (η_p_^2^) for ANCOVA and rank-biserial correlation (*r_g_*) for the Mann–Whitney U and Wilcoxon tests. Further information regarding data analysis is detailed in Section 4.

**Table 3:** Differences in expression of TLR7, TLR9, and BANK1 under the conditions of sleep deprivation, depending on sleep loss-induced mood changes.

		RE	NR	*p*-Value RE, Post-PSG vs. RE, Post-DS, Effect Size	*p*-Value NR, Post-PSG vs. NR, Post-DS, Effect Size	*p*-Value RE, Post-PSG vs. NR, Post-PSG	*p*-Value RE, Post-DS, vs. NR, Post-DS
After PSG	BANK1	45, −3.4 ((−3.8)–(−2.6))	27, −3.7 ((−4.1)–(−3.1))	0.329	0.021 *r_g_* = 0.44 *	0.104	0.799
After DS		47, −3.1 ((−3.4)–(−2.8))	29, −3.1 ((−3.5)–(−2.9))				
After PSG	TLR7	47, −0.8 ((−1.2)–(−0.5))	28, −0.6 ((−0.9)–(−0.3))	<0.001 *r_g_* = 0.65 *	<0.001 *r_g_* = 0.73 *	0.081	0.443
After DS		47, −1.5 ((−2.0)–(−1.0))	29, −1.3 ((−2.2)–(−0.7))				
After PSG	TLR9	45, 0.6 (0.2–1.0)	23, 0.45 (0.03–0.95)	0.731	0.715	0.580	0.983
After DS		47, 0.8 (0.0–1.2)	29, 0.65 (0.34–1.08)				
				*p*-value, RE vs. NR			
ΔBANK1		45, 1.1 (0.8–1.3)	27, 1.2 (1.0–1.4)	0.144			
ΔTLR7		47, 0.6 (0.3–1.0)	28, 0.4 (0.3–0.8)	0.691			
ΔTLR9		45, 0.6 ((−0.2)–1.1)	23, 0.9 ((−0.0)–1.3)	0.432			

Abbreviations: BANK1—B-cell scaffold protein with ankyrin repeats 1; DS—deprivation of sleep; NR—Non-Responders; PSG—polysomnography; RE—Responders; TLR7/TLR9—Toll-like receptor 7/9; Δ—post-DS value/post-PSG value. Data are presented as n, median (interquartile range, (IQR)) due to non-normal distribution. An asterisk (*) indicates the effect size, which was reported as rank-biserial correlation (*r_g_*) for statistically significant Wilcoxon test results.

## Data Availability

The raw data supporting the conclusions of this article will be made available by the authors on request.
